# “And I don’t know how I can protect him from everything that’s coming” - A qualitative study on minority stress among parents of transgender adolescents

**DOI:** 10.1186/s12888-025-06822-3

**Published:** 2025-04-24

**Authors:** Alexandra Brecht, Maryam Amacha, Judy Alan Richter, Sibylle M. Winter, Claudia Calvano

**Affiliations:** 1 Department of Education and Psychology, Clinical Child and Adolescent Psychology and Psychotherapy, Freie Universität Berlin, 14195 Berlin, Germany; 2 Department of Child and Adolescent Psychiatry, Charité-Universitätsmedizin Berlin, corporate member of Freie Universität Berlin, Humboldt Universität zu Berlin and Berlin Institute of Health, 13353 Berlin, Germany; 3 German Center for Mental Health (DZPG), partner site Berlin-Potsdam, 10117 Berlin, Germany

**Keywords:** Transgender adolescents, Parents, Minority stress, Qualitative research, Participatory research

## Abstract

**Background:**

Parents play a central role for transgender adolescents’ well-being and their coping with minority stress, like discrimination, rejection and violence. Yet, little is known about the experiences of parents facing minority stress as they support their transgender children who are either awaiting or undergoing medical transition. Therefore, we aimed to first examine minority stress experiences reported by the parents with respect to distal and proximal stressors. Second, we aimed to explore whether experiences relate to children’s stress experiences as secondary stress experiences, or if parents report minority stress directed at themselves as primary stress experiences.

**Methods:**

In the context of the participatory TRANS*PARENT study in Berlin, Germany, from April 2022 to September 2022 five focus groups were conducted with a total of 24 parents who have a transgender and/or non-binary child at the age of 11–18 years. Qualitative structuring content analysis was applied.

**Results:**

Parents reported distal stressors such as structural problems (in education, medical, psychiatric and psychological institutions, leisure activities and sports) and social rejection (peer rejection like bullying and violence, gaslighting, intersectionality, blame of others). Proximal stressors covered fears of future/anticipated discrimination, internalized gender stereotypes, and self-blame. Most experiences were secondary stressors, related to the child’s minority stress causing frustration and sadness for the parents, while especially fears of the future/anticipated discrimination, gaslighting and blame of others emerged as central primary stressors.

**Discussion:**

Results show a complex interaction of minority stressors affecting both parents and their transgender children on structural and social levels. The impairment of their child’s education and worries about its safety and life prospects burdened parents the most. The additional direct and primary stress experiences might impede their efforts to navigate their child’s transition process.

**Conclusion:**

In transgender health care, the multidimensionality of the structural and social impacts of marginalization needs to be addressed not only with respect to the transgender adolescents themselves, but also with respect to their caregivers. Further research should explore how child and parental stress interact and how family-based health care can alleviate stress on both child and parent level.

**Clinical trial number:**

Not applicable.

**Supplementary Information:**

The online version contains supplementary material available at 10.1186/s12888-025-06822-3.

## Introduction

An alarmingly high proportion of transgender^1^ adolescents report mental health problems such as depression, anxiety, self-harm and suicidality [1, 2]. They also face greater challenges with bullying and peer relationships than their cisgender peers [3, 4] and stigmatization is prevalent across many areas of transgender adolescents’ lives, within the family dynamics, in schools, and broader socio-cultural contexts [5]. To understand the impact of stigma on mental health among transgender people, the Minority Stress Model (MSM) [6, 7] defines distal and proximal stressors. Distal stressors are external stressors in the social and institutional environment and include discrimination, rejection, and victimization. Proximal stressors, on the other hand, relate to internal struggles such as internalized trans-hostility, fears of rejection, and concealment of gender identity. Resilience factors, such as community support, a sense of pride, and especially familial and parental acceptance help mitigate these stressors [8]. The associations between stressors and their outcomes within the MSM have been studied and confirmed by cross sectional qualitative and quantitative data among transgender persons themselves both for adults [9–11] and children and adolescents [8, 12, 13].

Research within the area of transgender adolescents underlines the important role of the parental and family context for their well-being in everyday life [14–16]. A large line of research deals with the role of specific parental behaviors and attitudes towards their transgender child. Studies consistently show that parental support and acceptance, demonstrated through behaviors and attitudes that affirm their child’s gender identity [17], correlate with better mental health outcomes for transgender adolescents compared to those that do not receive parental support [18]. This includes lower rates of depression, suicidal thoughts, PTSD, and eating disorders, alongside improvements in self-esteem and overall quality of life [16, 19, 20]. In line, transgender adolescents without parental support show higher suicidal tendencies [21, 22]. Further, parents play a major role for utilization of transgender health care, which can be either supportive or hinder their child’s social transition and access to gender-affirming healthcare, implying serious consequences for the child’s entire life [23, 24].

In addition to seeing parents as important agents in adolescents’ lives, we need to be aware that parents are confronted with their child’s transgender minority stress in everyday situations as well [25] and need to cope with challenges in their child’s social environment [14]. Existing research points to parental challenges related to everyday life in institutions like school and healthcare which often lack inclusive policies, such as proper use of names and pronouns, gender-neutral restrooms, and education about gender diversity [26, 27]. Many parents report that their transgender children have adverse experiences like discrimination by medical providers, by the extended family, and society at large [28] whilst parents also have difficulties obtaining information, particularly regarding medical transitions, and navigating through social systems in general [29] which can be termed as *secondary stressors*. Direct experiences of structural and interpersonal challenges and adversities, as follows termed as *primary stressors*, can impact the emotional wellbeing of the parents themselves. This is an area that has so far been largely neglected in research. Research in the field of child and adolescent mental health, mainly conducted among cisgender youth, shows that parents often experience considerable stress like burnout, emotional exhaustion, and even loss of control when parenting a child that is exposed to stress [30, 31]. Parents reported feelings of guilt and helplessness when they feel unable to support their children’s needs adequately, which in turn contributes to their own mental strain [31].

Until now, the MSM has not been applied to parents of transgender adolescents, and research on the specific stressors related to the marginalization of their transgender children and its impact on the parents’ well-being remains limited. Whilst acknowledging the explanatory power and importance of the MSM, researchers have called for extensions of the MSM regarding various minority groups, time, generational and political factors [32–34] which for instance has been addressed in terms of intersectionality by Rivas-Koehl et al. [34]. Other studies sought to explain minority stress not only as an individual but relational experience by analyzing minority stress in couples [35] and within the family [25, 33, 36, 37]. A meta-analysis literature review focused on minority stress on the family-level and found that children of LGBTQI + parents experience minority stress too. The authors state that minority stress is a family experience and can have an impact on the well-being of all family members even though they don’t necessarily belong to a minority themselves and therefore postulate a Family Minority Stress Model [38]. Only a few studies were conducted about the minority stress experiences of parents of transgender children. In a qualitative study (*N* = 40), parents of transgender prepubertal and pre-transitional (no medical transition steps) children under the age of 11 reported own experiences of distal stressors (discrimination, rejection, victimization and non-affirmation) and proximal stressors (internalized transphobia, negative expectations). In addition, parents reported negative psycho-social outcomes such as mental health distress (emotional burdens like feelings of loss, guilt, and anger, along with social isolation and disruptions to daily activities) [25]. Other studies showed emotional conflicts within the parental dyad concerning how to best support their child [28, 39] and that parents of transgender children often face negative social judgment for their parenting decisions and affirmative attitude towards their child’s transition [40]. So far, there is no known study analyzing minority stress among parents of transgender adolescents whose children are either pre-transitional (waiting for medical transition steps) or peri-transitional (already undergoing medical transition steps such as puberty blockers, hormone treatment or mastectomy). Especially in the German context where the launch of evidence-based consensus national treatment guidelines [41] has been accompanied by high controversies in the media and scientific communities [42], studies focusing on the affected families and their lived experiences are especially needed. Since parents play a key role in the context of their transgender child’s life, it is crucial to explore the lived realities by the parents themselves. This paper will focus on a qualitative exploration of the minority stress experiences in everyday life and across different settings among parents of transgender adolescents. Therefore, this focus group study aims to add important information and provide visibility and a voice to parents regarding their burdens.

## Method

### Study aim

The aim of the current study was to examine minority stress faced by parents of transgender pre- and peri-transitional adolescents and, in reference to Hidalgo and Chen [25] by analyzing (1) distal and proximal stressors and their impact (2) and whether the parents have experienced the stressors directly themselves (primary stressors) or towards their child (secondary stressors).

### Study design

The study uses data from the TRANS*PARENT project [43], a participatory research project involving transgender and non-binary youth and their parents. The project examines both the challenges, resources and needs of parents of transgender adolescents in designated focus groups. The current paper analyzes only minority stress expressed by the parents. A participatory approach was used because participatory research eliminates the typical division between “researchers” and “subjects,” involving community members in a collaborative manner that benefits and empowers them. Members of the transgender community, both adults and adolescents, as well as professionals in the field of transgender counseling constituted expert-groups before starting the project. Adult experts from the transgender community were involved in each research step, i.e. study design, selection of research aims, conduction of focus groups, data analysis and publication of the current paper. The study was conducted according to the guidelines of the Declaration of Helsinki and approved by the Institutional Ethics Committee of the Charité-Universitätsmedizin Berlin (EA2/275/21, date 17 December 2021).

### Procedure

Written consent was obtained by each participant. The parents informed their children about their participation in the study whilst most children participated in their own focus groups which was part of another study within the TRANS*PARENT project. Focus groups for parents of transgender adolescents (aged 11–18, identifying as transgender and/or non-binary) were held for the joint exploration of parental experiences. Before participating in a group session, each parent was asked whether they had a topic they would like to talk about in the group. The topics mostly convered the desire to have an exchange about the challenges of raising a transgender child and where to get support from. A semi-structured interview guide (which can be retrieved from supplementary material S1), covering gender identity, stress, resources, and needs, was used. The guide included 29 questions and was adapted for each group considering the participant’s concerns. The questions targeted experiences of both parents and their children and covered various areas such as gender identity, gender dysphoria and transition as well as areas of experiences within family, friends, school and other institutions, the general health care system (e.g.: “What do you think are the biggest problems regarding your child’s trans identity in your broader family?”; “Have you faced any challenges with healthcare institutions?”); and more general questions about problems and stressors (e.g.: “What have you identified as the greatest burdens so far?”; “If you’re sad and stressed, where do you go?”). The focus groups were moderated by the same clinical psychologist (White, cisgender female, A.B.) with experience in the transgender field, conducted in person and online between April and October 2022, lasting about 1 h and 43 min on average, ranging between 95 and 113 min (see Table 1). Focus groups were conducted in German since all participants were German-speaking. Live groups took place at Charité-Universitätsmedizin Berlin, online groups were held via Microsoft Teams and all focus groups were recorded whilst a member of the research group took notes. Sociodemographic data were gathered before participating in the focus groups via a questionnaire, which included questions about parental age, gender, nationality, and family status. The survey also assessed the extent of parental support given to the child (“How would you describe the level of support for your child?”), using a five-point scale for both items ranging from 1 = very low to 5 = very high. For this publication, all questionnaires were translated to English and can be retrieved from Open Science Framework (OSF) [43]. Participation was compensated with 50€. At the end of each focus group, parents were encouraged to stay connected in group chats.

**Table 1:** Overview of participant and group characteristics within each focus group

	Group A (*n* = 6)	Group B (*n* = 4)	Group C (*n* = 5)	Group D (*n* = 6)	Group E (*n* = 3)	Total
Female (n)	3	3	4	3	2	15
Male (n)	3	1	-	3	-	7
Non-binary (n)	-	-	1	-	1	2
Duration (minutes)	109	95	113	101	100	518
Setting	live	live	online	live	online	

### Sample recruitment

Participants were recruited via the interdisciplinary special consultation for questions of gender identity in childhood and adolescence (German abbreviation GIF), affiliated to the Social Pediatric Center of Child and Adolescent Psychiatry at the Charité–Universitätsmedizin Berlin and via an advertisement on its website. The GIF is specialized in the affirmative care of children and adolescents with gender incongruence, with high relevance given to the inclusion of caregivers as well as the recognition of the individual development stage of each person. Inclusion criteria was being a caregiver of a transgender child/adolescent, exclusion criteria were an acute psychotic episode, revocation of consent, acute psychological distress requiring a different form of therapy (e.g., inpatient care) and severe intellectual disability. Families were approached by the responsible psychotherapist and clinical psychologist of the GIF who in the initial session had given their consent to be contacted for research, who had expressed interest in exchanging with other parents or who showed interest in the study after seeing a flyer distributed during consultation in the practice.

### Data analysis

**Fig. 1:**
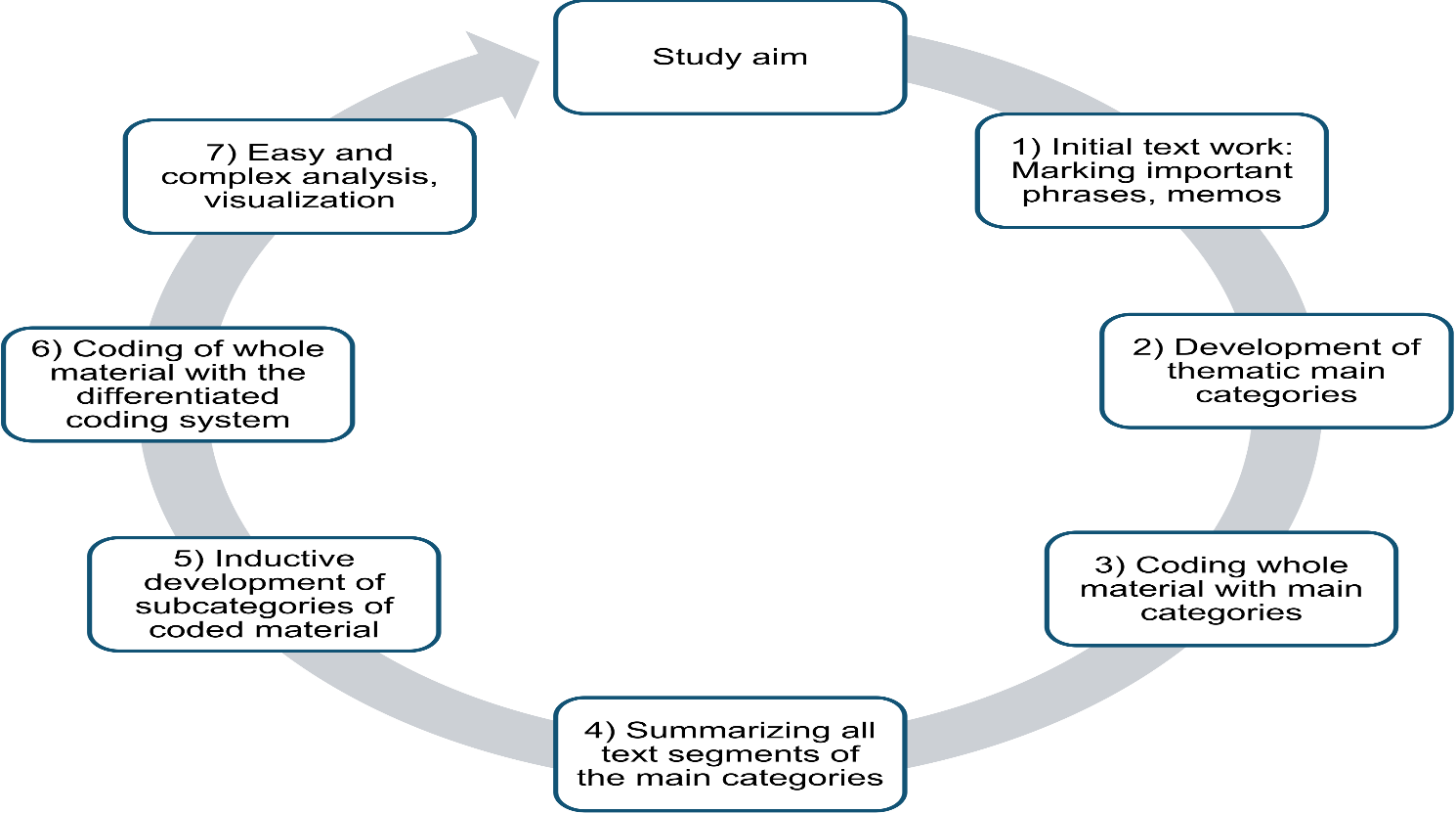
Process of qualitative structuring content analysis (QSCA) aligned to Kuckartz [46]

A qualitative approach was chosen to explore the challenges faced by parents of transgender youth. Audio recordings of focus groups were transcribed using Trint software [44]. Data were analyzed using qualitative structuring content analysis (QSCA) in MAXQDA software [45], following Kuckartz and Rädiker’s methodology [46]. This method involves systematically organizing and interpreting textual data by coding and categorizing themes to identify patterns and meanings. It emphasizes an iterative process where researchers refine categories and interpretations through a repetitive cycle of continuous comparison and the development of a conceptual framework. The respective work process is displayed by Fig. 1. The main categories distal and proximal stressors were deductively derived from the minority stress model. Then a mixed approach on a rather descriptive level was chosen. One starts with a category system consisting of relatively few categories, which are derived from the respective theory about minority stress among transgender people. These initial categories are only used as a starting point and function as a kind of search framework, meaning the material is examined for the occurrence of relevant content and roughly categorized. In the second step, subcategories are then developed inductively through multiple cycles of interpretation, considering only the material assigned to the respective main category [46]. An intercoder reliability (agreement of the coding by both raters in the final code system) check was excellent, showing an agreement of 86.49%. Categories were refined in collaboration with a community-based counseling network Trans-Kinder-Netz e.V. and experts within the Charité working group. The final system included 69 categories, with eight main categories and 61 subcategories, totaling 945 codes. For the current paper, only the main theme minority stress was included in the analysis. Notably, a category could have already been created based on a single answer.

### Study sample

In total, we contacted 36 families from the GIF, and four families had contacted us themselves via e-mail because of the internet advertisement. From these 40 parents, 12 declined participation and four couldn’t arrange the focus group’s meetings, leading to *N* = 24 parents participating. Descriptive data can be retrieved from Table 2. Five focus groups were held which included between 3 and 6 parents each. Composition and setting of each group are displayed in Table 1.

**Table 2:** Descriptive data of parent sample and their transgender children

		Parents (*n* = 24)	Adolescents (*n* = 19)
Age in years, M (SD)		49.9 (7.4)	15.42 (2.01)
Gender identity, n (%)	Female	15 (62.7)	2 (10.5)
	Male	7 (29.2)	14 (73.7)
	Non-binary	2 (8.3)	3 (15.8)
Race/ethnicity, n (%)	White	15 (62.5)	15 (62.5)
	Asian	1 (4.2)	1 (4.2)
	Multiracial	1 (4.2)	1 (4.2)
	No answer	7 (29.2)	7 (29.2)
Migration history/country of origin	Yes	5 (20.8)	-
other than Germany, n (%)	No	15 (62.5)	-
	No answer	4 (16.7)	-
Relationship status, n (%)	Married	11 (45.8)	-
	Divorced	7 (29.2)	-
	Separated	1 (4.2)	-
	No answer	5 (20.8)	-
Monthly income, n (%)	< 500	2 (8.3)	-
	500–1000	1 (4.2)	-
	1000–2000	2 (8.3)	-
	2000–3000	6 (2)	-
	> 3000	6 (25)	-
	No answer	7 (29.2)	-
Medical transition status, n (%)	None / waiting	-	8
	Puberty blockers	-	2
	Gender affirmative hormone treatment	-	9
	Mastectomy	-	1
Support of child, n (%)	Very low	0 (0.0)	
	Rather low	1 (4.2)	-
	Not sure	3 (12.5)	-
	Rather high	8 (33.3)	-
	Very high	6 (25.0)	-
	No answer	5 (20.8)	-

## Results

**Fig. 2:**
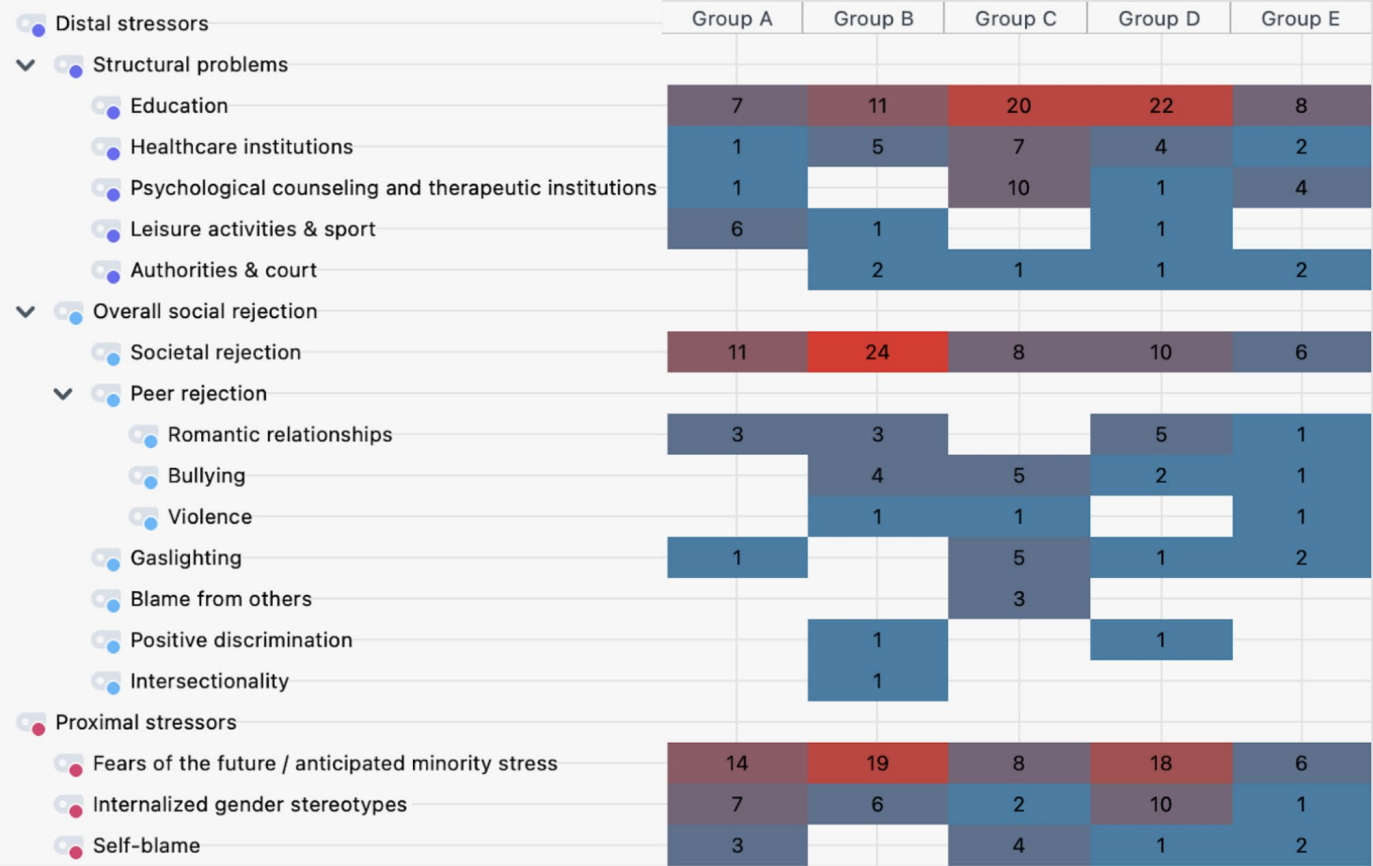
Visualization of the frequency of mentions of each code among the focus groups

In the following, results of the theme minority stress separated in categories of distal and proximal stressors are presented. Figure 2 provides an overview of the results of QSCA for the category system of both distal and proximal stressors including the subcategories and their frequency of mentions summed for each focus group. In the following, results for distal and proximal stressors will be presented. For easier comprehension, the subthemes were summarized in key points style. For each category, one quote (translated to English by the first author, A.B.) is included to help characterize particular topics.

**Fig. 3:**
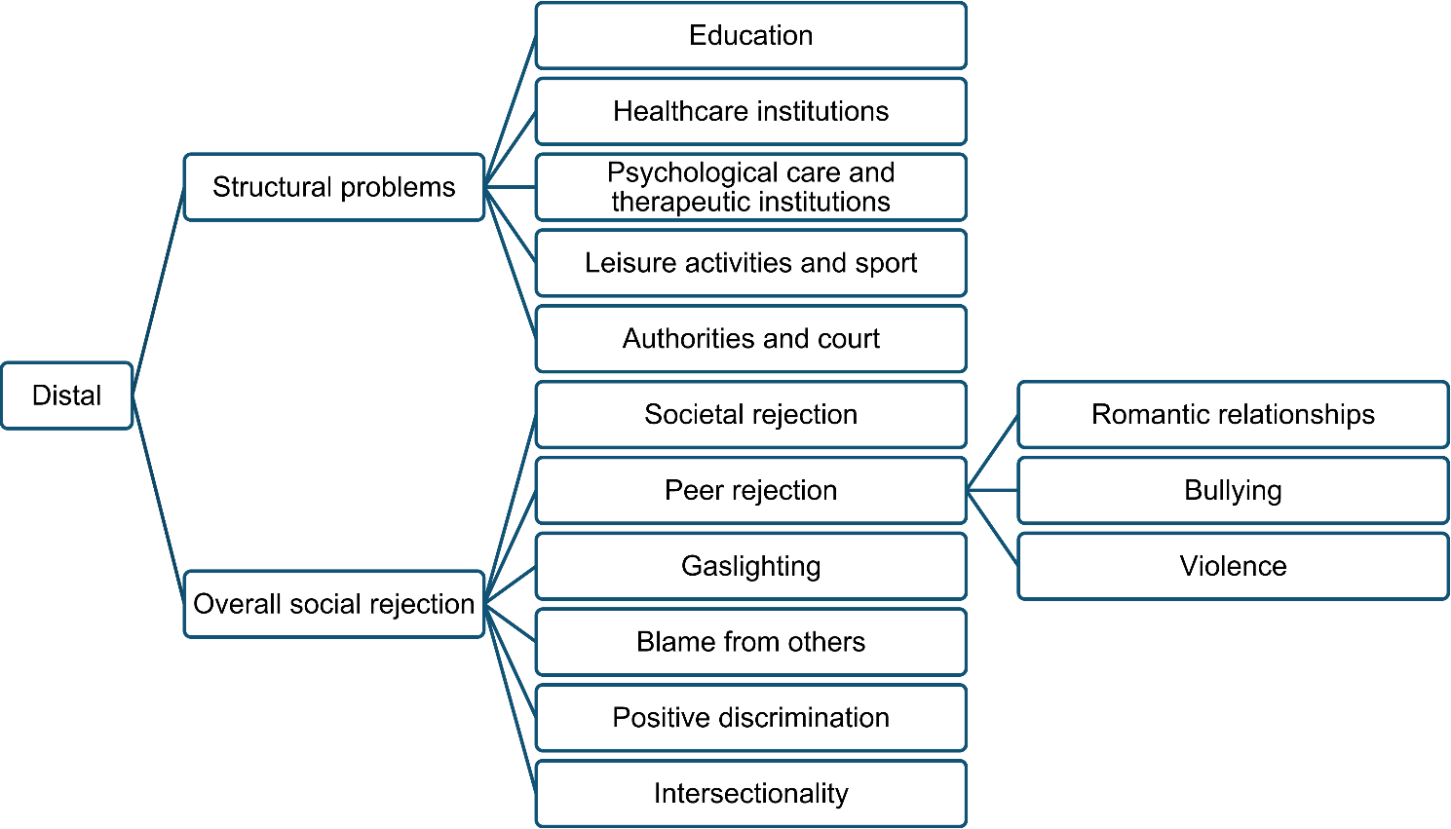
Overview of categories and subcategories for distal stressors

### Distal stressors

Analyses revealed both distal stressors experienced by the child in the perception of the parents (secondary stressors) and stressors directly experienced by the parents (primary stressors). Subcategories within the distal stressors cover aspects of structural problems (5 subcategories) and overall social rejection (6 subcategories) which are displayed in Fig. 3. Results will be presented in order of their importance as defined by the frequency of reports.

#### Structural problems

This category covers burdens that are perceived in contact with institutions and employees of these institutions. Rejection of the child’s gender incongruence by institutions manifested in discrimination, stigmatization, access barriers and trans-hostile structures. Trans-hostile structures mean circumstances and conditions in the environment that actively exclude transgender people. Responses were divided into five subcategories which mirror the structural problems specific to areas of everyday life: Education, healthcare institutions, psychological institutions, authorities and courts, and leisure activities and sports.

##### Education

The category *education* includes the challenges faced by children with gender incongruence in school and secondary education that have an impact on the parents. The content of this subcategory covered five main themes:

- Facilities: Ten parents described difficulties with toilets and changing rooms, often resulting in children avoiding these facilities altogether. When special accommodations were made, such as using disabled or staff toilets, it made the children feel isolated. These conditions triggered emotions ranging from frustration, anger and fear to despair in the parents. “*It’s an incredibly big problem to make unisex toilets. So*,* my child just doesn’t go to the toilet at school… And then there are no individual changing rooms*,* only group ones. It’s really difficult.*” (group E, item 138).
- Sports and physical education: Three parents experienced stress and anger due to a lack of proper guidelines for physical education, and two parents highlighted specific struggles with swimming classes. One parent had to endure that their child was even excluded from physical education due to inadequate arrangements. *“In the end*,* we could only resolve it by having [name] get a sports exemption from the pediatrician because it just wasn’t feasible. It wasn’t reasonable to expect otherwise.”* (group C, item 97).
- Negative reactions and bullying: Both teaching staff and peers sometimes reacted negatively to a child’s coming out, which was shocking to the three parents. Bullying by classmates and even teachers was reported by five parents, with one case involving physical violence which made parents feel of anxious and helpless. One parent faced challenges in finding a school that would accept their child. Another experienced outright rejection from a teacher, leading to intervention from the administration. *“Well*,* at our school*,* it was a bit traumatic somehow … It was just… my child felt like*,* ‘I’m weird*,* I don’t fit in here.’ They were expecting huge problems and difficulties. It was really totally unpleasant … They were just seeing problems—like*,* how do you use the bathroom? Where? And how will it work in gym class? Just creating this sense of fear. I found that unbearable.”* (group A, item 82).
- Use of name and pronouns: Two parents felt anger as teachers and pupils refused to use the child’s chosen name and pronouns, and one mentioned ongoing discriminatory behavior from staff. Two parents expressed their frustration about school documents still being issued in the child’s birth name and gender, causing stress for the parents since they have to confront the schools constantly and making themselves vulnerable at the same time. *“Um*,* but of course*,* the certificates are all in the name of [name]… And I always have this conflict.”* (group C, item 83).
- Negative impact on future education: Four parents had to worry permanently because the psychological stress of gender incongruence led to frequent absences from school. Two parents experiences high distress due to school dropouts and changing schools, resulting in learning gaps that impacted academic performance. One parent expressed concerns about their child’s application for professional training, fearing discrimination during the hiring process. Another worried about the potential rejection their child might face when starting university in a different city. *“To be quite honest. In a small town*,* I think it’s much more intolerant and that’s why we naturally thought a bit about where he goes for studying. That will now be [name of big city]. And we just hope that you can live there the way you want to live.”* (group A, item 81).

##### Health care institutions

Within the category *health care institutions*, stressors experienced by parents in their interactions with general healthcare facilities, professionals, and the care provided to their transgender children are summarized into seven main themes:

- Difficulty in accessing overall medical care: Three parents had significant struggles in accessing gender-affirming medical and psychiatric care for their children because specialized centers had long waiting times or had even stopped accepting new patients which made the parents desperate. In one case, care was refused due to the child’s age, another parent had to endure that care was refused even in urgent situations like suicidal tendencies. One parent expressed confusion about inconsistencies between outpatient and inpatient care, noting that no close supervision was provided during inpatient treatment. *“And also these waiting times … To open up now*,* to get help and then to wait again and tell the child: ‘So you know*,* we’ve been postponed’. I was sorry too. And then I also told the doctor on the phone*,* ‘You know*,* that it’s not possible*,* that we have to wait again now’. Yes*,* and that’s how we’re dealing with it now*.” (group A, item 186).
- Lack of expertise in gender incongruence: One parent expressed frustration and helplessness about the lack of expertise among healthcare professionals when dealing with gender incongruence. *“Although I have to say*,* unfortunately they weren’t really helpful at [name of clinic] either*,* I thought. It was a very young doctor and he didn’t really know what we should do now.”* (group C, item 35).
- Inappropriate handling by doctors: Two parents were upset about instances where doctors questioned their child’s gender identity during unrelated medical exams, causing embarrassment. Another parent was shocked that their child’s expressed gender identity was disregarded during treatment. *“The pediatrician … behaved completely absurdly. I told her that again in private. It was really terrible. It was just about…giving a vaccination. And I had said beforehand*,* “Please just call them [name of child]. They want to be a boy.” And then she really lifted the shirt and examined his chest. At that time*,* we didn’t have a binder yet. [Name of child] almost sank into the ground*,* and I felt so sorry for him. And then she asked*,* ‘Are you already in puberty? Do you already have hair under your arms? Do you already have hair down there?’ And I thought*,* ‘Wow. He wouldn’t even tell us that. It’s such a private thing.’ He locks himself in the bathroom and everything. And I thought*,* ‘Wow’. I stood there and thought*,* ‘This can’t be real.’ I was so stunned I couldn’t even react and stop her somehow.”* (group D, item 167).
- Logistical and structural issues in hospitals: One parent was frustrated by the absence of gender-neutral hospital rooms, despite assurances, leading to the child being placed in a room aligned with their assigned sex at birth. This resulted in the parent discontinuing the treatment. *“And I find that extremely frustrating because I’ve seen that it really depends on who we end up dealing with. And*,* well*,* it’s actually a support system that very often excludes people.”* (group C, item 85).
- Misgendering and deadnaming: Two parents shared that they were annoyed by repeatedly providing their child’s chosen name and pronouns to prevent uncomfortable situations during treatment. *“We have to say at every practice*,* please write ‘man’*,* so that he is not addressed as ‘she’.”* (group B, item 64).
- Challenges with gynecological treatment: Five parents were indignant about gynecological care, with one child being denied treatment due to not a being cis woman, while others felt uncomfortable and had to justify their appearance. *“But at some point*,* he must have another check-up with the gynecologist. Then he’ll have to explain himself*,* which is bad enough. Thank God it’s still mandatory to wear a mask*,* so you might not even see it yet. Like I said*,* sitting bearded in the gynecologist’s waiting room without a woman. That’s another situation we have to get through.”* (group D, item 318).
- Loss of trust in healthcare system: One parent reported that they and their child had lost trust in the healthcare system, leading to a reduced use of medical services and even more responsibility to the parent. *“Although I think there were some moments when he really should have gone to the clinic*,* but he refused because he didn’t have trust*,* which I can understand based on those experiences. And so*,* I had to carry that burden alone at home*,* really.”* (group C, item 85).

##### Psychological counseling and therapeutic institutions

The category *psychological counseling and therapeutic institutions* displays stressful experiences reported in contact with psychotherapeutic practices, with facilities, the staff of these institutions and the care provided within them. It covers the two identified areas:

- Difficulty accessing affirmative psychological care: Two parents were suffering from long waiting periods before their children received psychotherapeutic treatment because they were scared for its mental health. Four parents found the search for suitable psychotherapists to be lengthy and stressful. *“We have now found a therapist like that. But as I said*,* it took forever and there were many waiting lists. Difficult to find. It would have been nice if we’d had someone like that right from the start.”* (group C, item 129).
- Non-affirmation: Three parents reported a lack of trust due to situations in which acceptance was initially expressed by psychotherapists, which then turned out to be rejection or a lack of knowledge. Two parents had to be suspicious and alert by the psychotherapist’s attitude toward their child’s gender incongruence, which affected the therapy experience. One parent was upset that psychotherapists appeared overwhelmed when caring for individuals with gender incongruence, leading to incorrect decisions or referrals. *“A psychologist who was supporting him and always acted super understanding and all that*,* but then in a parent meeting said something like*,* ‘Well*,* I don’t believe that exists at all’ and that [name of child] is just confused*,* that it’s just a phase*,* and that I shouldn’t take it all so seriously. But to [name of child] directly*,* she always said*,* ‘It’s all fine*,* go ahead*,*’ and always listened. And I find that really difficult … Yes*,* it tears me apart. It completely destroys me … I’m always in this position where I have to decide: Do I tell the child*,* ‘Well*,* the therapist told me this and that?’ Or do I handle it diplomatically? Do I just say*,* ‘Let’s look for a new therapist?’.”* (group C, item 91).

##### Leisure activities and sport

Recorded stressors experienced during the adolescent’s free time activities were summarized in the category *leisure activities and sport* by two main themes:

- Concerns about gender binary in teams: Two parents are concerned that there is a lack of regulation on how the sporting performance of transgender people should be measured and evaluated. Two parents constantly worried that their child would no longer be able to play their sport in their previous sports team after coming out as transgender, as their gender identity no longer matched the gender-specific team. *“It’s also a predominantly Turkish Muslim soccer team. And yes*,* he’s* [trans male] *afraid that he’ll be kicked out if he’s not a real girl. Then he won’t be allowed to play in the girls’ team. He really likes playing there.”* (group A, item 94).
- Concerns about gender binary in facilities: Two parents expressed anger that there were problems with gender-neutral facilities, such as changing rooms and toilets, which made it difficult to play sports. *“I was really worried. The child has been swimming since he was five. Now he was like ‘I don’t want to go to the swimming pool anymore*,* there are only men or women and changing rooms’.”* (group A, item 8).

##### Authorities and court

The category *authorities and court* summarizes stressful experiences reported in contact with authorities, courts, their staff, and the services provided by these institutions within two themes:

- Inability to change gender entry: Two parents reported that the staff at the municipal office was not prepared to handle transgender individuals, particularly transgender youth. One parent described that municipal office staff were unfamiliar with bureaucratic procedures and reported feeling that there were no clear guidelines, leaving the parent with the impression that the institution could not help. Stress was reported by two parents due to complicated legal processes for name and gender changes, reflecting broader societal discrimination. Further frustration was expressed over the slow progress in changing laws that could facilitate the transition process. *“So we applied for a passport*,* and then I asked*,* um*,* what we could do about the gender entry*,* because*,* you know. And they were completely overwhelmed; they knew nothing. They didn’t know what options existed. That shocked me*,* you know. I thought*,* okay*,* do I have to brief you on what it is and what’s possible? And I found that striking. I also noticed that it was very uncomfortable for my child. Sitting there*,* and suddenly the people were running around like headless chickens or something.”* (group B, item 52).
- Dependence on expert opinions: One parent shared the experience of a court-ordered psychological expert opinion which did not support the child’s transition, and the parents felt intimidated to raise objections for fear that the court might rule against the transition. One parent noted that bureaucratic procedures in authorities and courts required that the parent and the child had to justify themselves repeatedly. Parents stressed experts’ gate-keeping position as they were heavily reliant on the acceptance and support of the staff, which could not always be assumed. *“Everything changes and the next step*,* so to speak*,* in the German system is this change of civil status name. And for that I need two certificates. And that means that if my child starts taking hormones to be a man*,* I don’t understand how he can be denied this name change and also this masculinity. I could really get into it*,* because it’s such an issue of discrimination.”* (group E, item 66).

#### Overall social rejection

This category covers various fields of distal stressors in the context of social rejection. The following subcategories were found: societal rejection, peer rejection (with further subcodes of romantic relationships, bullying, violence), gaslighting, blame from others, positive discrimination, and intersectionality.

##### Societal rejection

In this category, burdens that are perceived because of the social positioning of transgender people, hurdles, stereotypes, political attitudes and developments, laws and social discrimination were coded as well as cross-references to other subcodes of social rejection that could not be separated clearly, which made this code the largest of the system. Themes that had no cross-references to other subcodes were the following:

- Stress from binary system: Four parents found the binary gender system stressful, as it limits recognition of transgender identities and lacks gender-neutral spaces. One parent described a phenomenon where initial acceptance of a child’s gender incongruence can quickly turn into rejection if they do not conform to binary norms. *“And I’m worried about how society will react to this [transgender identity]. How the environment reacts to it*,* how binary the whole world is. That strikes me. In every museum toilet man*,* toilet woman. I feel the pain of my child. Having to make a decision where you don’t feel like you belong anywhere.”* (group A, item 153).
- Cultural acceptance: One parent observed greater acceptance of their child in cultures that are less strictly binary compared to Western culture. One parent was bothered by the irrationality that homosexuality is illegal in their country of origin, whilst trans identities are accepted but only if surgery has been performed. “*Sometimes I think*,* maybe she would be more understood by others or feel more comfortable in this world. Such as with these games and these mangas that she reads about and follows closely*.” (group A, item 53).
- Negative political climate: One parent raised concerns about a worsening political climate regarding societal acceptance of transgender individuals. One parent noted that negative generalizations from individual cases contribute to widespread social rejection of transgender people. *“That’s absurd*,* yes. It’s such a strange discourse and then society jumps on it*,* the newspapers all pick it up and it scares me. I’m afraid that the*,* shall I say*,* pleasant climate that we might now have for our children could quickly change.”* (group B, item 143).
- Acceptance issues for parents: One parent reported that societal norms make it difficult for parents to accept their child’s transgender identity. *“Um*,* and I think that’s why I was sometimes so worried and almost wished that he wasn’t transgender so that he wouldn’t have to go through it somehow.”* (group C, item 49).

##### Peer rejection

The category *peer rejection* covers real or feared rejection by (potential) romantic partners, friends, classmates and other peers of the child. The rejection may take the form of teasing, exclusion or even bullying and violence.

###### Romantic relationships

The category romantic relationships covers parents’ reports about their children’s experiences with partnership, sexuality and dating.

- (Fear of) rejection by romantic partner: One parent reported that the child had experienced a romantic relationship being ended by the other person after finding out about the child’s transgender identity. One parent found it distressing that the child avoids relationships due to concerns about being rejected by romantic relationship partners due to their trans identity. *“Well*,* he doesn’t have any sexual relationships or relationships yet. Romantic relationships*,* I’d say. I think I’m even more worried about that than him.”* (group D, item 405).
- Avoidance of intimacy due to gender dysphoria: Three parents reported that the child currently does not want to get involved in romantic relationships due to the psychological stress caused by gender dysphoria and dealing with their own body and transition measures, which made the parents sad. “*He’s very clear about that. He just knows that he won’t have that at the moment like others. Like the first times [kisses*,* sex]. It’s totally sad*.” (group D, item 197).

###### Bullying

The category *bullying* included parents’ reports about their child being ostracized and bullied in their social group due to being transgender.

- Bullies: Five parents were devastated about the bullying of their child by young people of the same age. Another parent felt especially desperate that bullying had been carried out by both classmates and teaching staff, resulting in the parent reporting the incident. One parent reported that their child had changed schools due to a “homophobic incident”. *“So we have really bad*,* bad*,* bad school experiences with the worst kind of bullying*,* including from teachers*,* and we also reported a school to the Senate.”* (group C, item 17).

###### Violence

The category *violence* records the stress experienced by parents as a result of the child experiencing physical violence from people in their social environment due to belonging to a marginalized group.

- Violence against other trans people: Two parents were scared because they had heard of other transgender people who had experienced physical violence from other people due to trans-hostility. *“I have to say*,* I’m really very afraid of that. I recently saw a report on television mentioning that*,* I believe*,* one in ten transgender people has been a victim of violence. And then there’s all the online bullying that can start under certain circumstances. I’m really terrified of that*,* I must admit.”* (group E, item 73).
- Violence against their own child: One parent reported that the child had experienced physical violence from classmates of the same age after coming out as transgender. *“After the summer vacation*,* things got really bad. People were very hostile*,* he was physically attacked*,* he was psychologically attacked*,* he was threatened. So*,* it escalated to the point where he almost attempted suicide. I found the farewell letters just in time and was able to stop everything. Um (pause) He was not admitted to the clinic. We were sent home again*,* even though*,* in my opinion*,* I had a very suicidal child sitting in front of me. And I had to take him home again*,* which completely overwhelmed me.”* (group C, item 49).

##### Gaslighting

The category *gaslighting* examines repeated questioning or undermining of the child’s gender identity towards the child and the parents (Riggs & Bartholomaeus, 2018). Gaslighting functions as a form of psychological abuse that undermines victims’ sense of reality (Sweet, 2019).

- Gaslighting of both child and parent: Six parents reported gaslighting towards the child and themselves by other people in relation to the child’s gender incongruence. Outsiders denying them and their child the ability to assess whether the child’s feelings are actually gender incongruent was a stressful experience for the parents. *“Some people*,* even within our own family*,* still say things like*,* “[name of parent]*,* be careful. What if this is just a trend? … Maybe your child is only identifying this way because they’re struggling to make friends and just want to belong somewhere.”* (group A, item 27).

##### Blame from others

This category contains the stressful experience of other people blaming the parents for the child’s gender incongruence, their psychological stress or reproaches from other people regarding support for the child’s transition.

- Parents are blamed: Two parents reported experiences of being blamed by other people, particularly by people in care institutions. They reported that outsiders blamed the parents for being responsible for their child’s psychological stress whilst neglecting external social influences. *“And it was also a lot about what a wrong position I might have as a mother*,* that he*,* that he’s like this. Um*,* which I think is fine to look at. But unfortunately*,* up to a certain point*,* uh*,* the world has a lot of influence and not just me.”* (group C, item 49).

##### Positive discrimination

The category *positive discrimination* displays experiences of parents when other people emphasize their child’s transgender identity as particularly positive and but still being different or even “exotic” (*othering*).

- Othering: Two parents described it as stressful that other people emphasized the child’s gender incongruence to them as particularly positive, as the parents perceived this as discrimination against the child. One parent reported that medical professionals expressed joy at being able to treat a transgender child. The other parent emphasized the child’s normality, which was denied to the child when other people described gender incongruence as something particularly great. *“Well*,* in my circle of friends*,* there was a lot of interest too. I often heard a phrase that started to bother me after a while: ‘Oh*,* that’s great.’ Of course*,* my child is great*,* but most of all*,* they’re just normal. It’s not something unusual or extraordinary.”* (group B, item 44).

##### Intersectionality

The category *intersectionality* captures the parents’ stress caused by the child’s experiences of multiple discrimination due to belonging to several marginalized groups (Crenshaw, 1989; Toomey et al., 2017).

- Multiple marginalized identities: One parent was resigned by the stress of discrimination experienced by their child due to belonging to a gender minority, a sexual minority and to a minority due to their child’s migration background. *“My son was discriminated against because he is a foreigner*,* because he is transgender*,* because he also said at the beginning ‘Yes*,* I am homosexual’ and so on*,* many different minorities.”* (group B, item 151).

### Proximal stressors

**Fig. 4:**
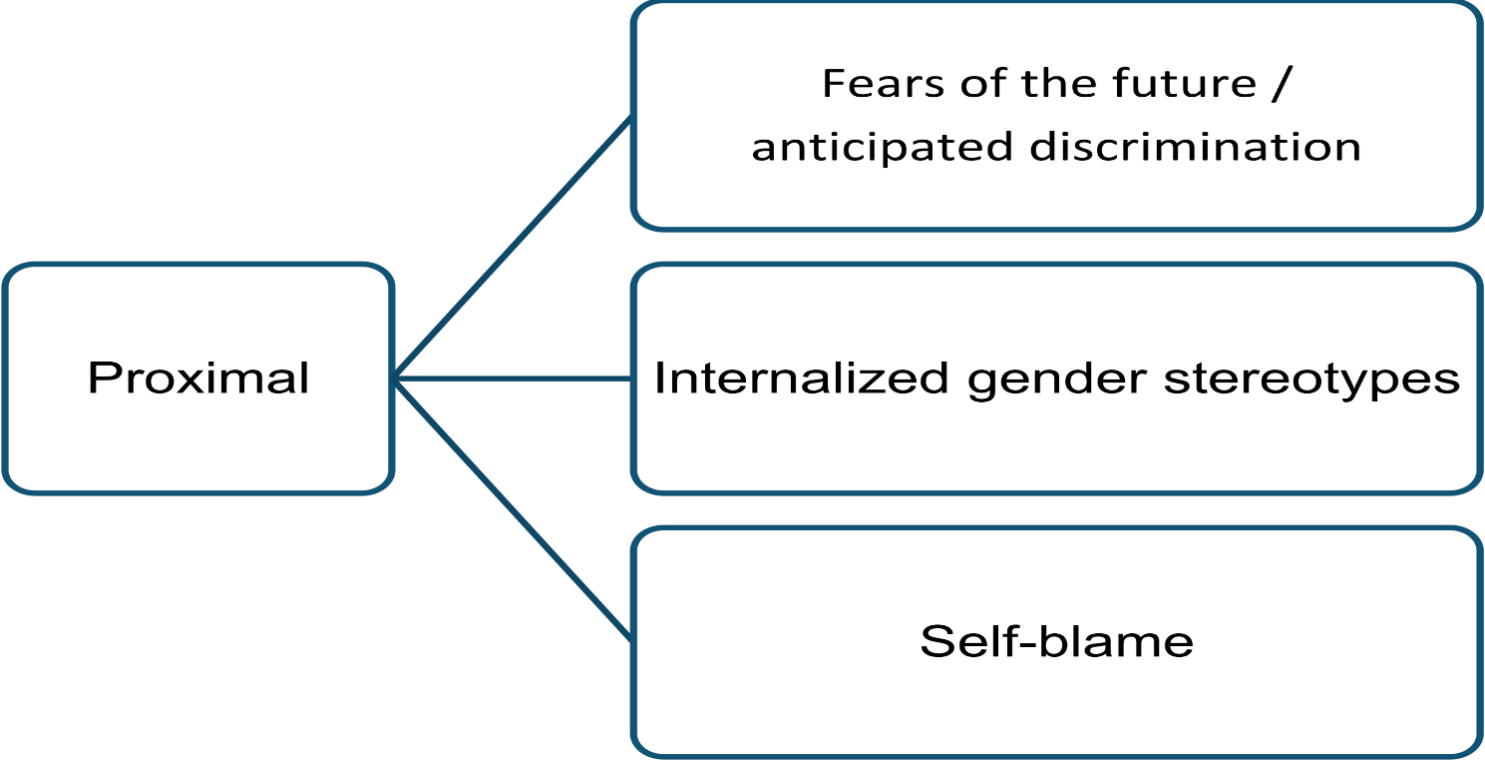
Overview of categories and subcategories for proximal stressors

This subordinate category captures internal stressful experiences of the parents due to the child’s minority status, which can manifest themselves in the form of internalized prejudices against gender minorities and negative perceptions of the future [7, 25]. Three subcategories were found: fears of the future/anticipated minority stress, internalized gender stereotypes and self-blame which will be presented by descending frequency of mentions as displayed by Fig. 4.

#### Fears of the future/anticipated minority stress

This category records the parents’ stress caused by negative perceptions of the child’s future due to gender incongruence. Nine themes were found:

- Concern about protection from negative experiences: Five parents expressed concern that they would not be able to protect their child from the negative experiences they might face due to belonging to a marginalized group. *“And that actually was*,* when he came out*,* I think my biggest feeling was that I thought*,* oh no*,* oh my God*,* he’s going to go through hell*,* and I don’t know how I can protect him from everything that’s coming.”* (group C, item 49).
- Doubt about a happy life: Six parents feared that the child would have a difficult life and would be happier if they conformed to cisnormativity. *“So*,* for me*,* I also had the idea that he could have it so much easier in life. I know that’s so hetero normative.”* (Focus group D, item 405).
- Psychological stress: One parent expressed concerns that their child would experience significant psychological stress due to discrimination and the daily struggles of meeting others’ expectations. One parent expressed concerns that the child lacked self-confidence to deal with the challenges of belonging to a marginalized group. *“He’s just not really well. I’m constantly worried. He says he’s not thinking about suicide. I believe that too. But still this instability*,* that’s it.”* (group A, item 139).
- Fear of discrimination and social rejection: Five parents feared that their child would be negatively perceived and rejected by their social environment. Three parents feared that the child could become socially isolated due to rejection, also regarding partnerships, which could lead to severe psychological stress. *“But it was definitely a wild ride for me*,* because my fears that I wouldn’t be able to protect him came true*,* unfortunately in many contexts.”* (group C, item 49).
- Fear of safety: Eight parents feared that the child might experience bullying, as well as physical and sexual violence. *“And we’re just really afraid of physical*,* emotional*,* or maybe even sexual assaults if he somehow comes out after all.”* (group D, item 177).
- Concerns about gender acceptance: Two parents worried that their child would not be fully accepted as either male or female due to their gender incongruence. One parent also worried that their child won’t ever pass as a cis man even after transition. *“I’m really curious to see how it’s going to turn out. And you also have to be prepared for the possibility of being rejected—that you won’t be accepted by the boys*,* the group you feel more aligned with*,* but also not by the girls*,* where you were before. I think that’s pretty tough to deal with*,* or however you want to put it.”* (group B, item 76).
- Concerns about independence and future: One parent was concerned that the child may not be able to live independently, and two parents expressed general anxiety about the child’s uncertain future. *“And we also hope that when he has arrived in the right body at some point*,* that his eating habits will gradually normalize. That at some point he will be in a position to take up a job and lead a regular life. In other words*,* to become independent of parents in the end. But I think that just needs a bit more time.”* (group C, item 53).
- Workplace discrimination: Four parents worry that their child might face professional disadvantages or discrimination due to their gender incongruence. One parent expresses ambiguous feelings about being afraid of discrimination but also looking forward to their child’s progression: *“So*,* he will probably start an apprenticeship in August*,* if all goes well*,* which will certainly bring new challenges. How do you do that then? How will the care and treatment continue there? Will it all work? These are the fears I have now*,* but they are also positive somehow.”* (group C, item 97).
- Concealing gender identity: One parent hid the child’s gender incongruence from the family due to fears of rejection in the family’s country of origin. Another parent reported mental health issues because they had to keep the coming out a secret. *“I have to say*,* unfortunately*,* I have a mental illness—a bipolar disorder—which means I sometimes have to go to the hospital when I’m in a phase. And the immense psychological pressure of not being allowed to talk about it triggered a manic episode for me*,* and I sadly ended up in a psychiatric hospital.”* (group C, item 35).

#### Internalized gender stereotypes

The category *internalized gender stereotypes* analyzes the stress experienced by parents due to the fact that the child cannot be classified in the binary system, i.e. does not clearly assign itself to the female or male gender.

- Internalized gender binary: One parent reported it as stressful that the child could no longer be categorized in the binary social system after coming out as transgender and non-binary. One parent described perceiving the internalized gender binary as a burden as well as the feeling of loss. “*But that was*,* at the beginning I thought*,* I’m totally crazy*,* this can’t be true. Well*,* because I’m bisexual myself*,* I lived as a lesbian for a very long time. I always thought I was the most liberal person in the world when it came to that. And it shocked me so much*,* I cried so much*,* just for my daughter somehow*.” (group C, item 134).
- Desire for certainty: Another parent expressed confusion that their child did not exhibit male stereotypical gender expression after coming out as trans male, for example, wearing stereotypical female clothing or painting fingernails and dyeing hair pink. *“Sometimes it can be a bit crazy. But I also think I just felt like saying*,* okay*,* either we go with the classic option where he’s a girl now*,* so we’ll do pigtails and blonde*,* or he’s a boy*,* which works too. Just make up your mind*.*”* (group D, item 223).

#### Self-blame

This category displays the stress parents felt when blaming themselves for their child’s gender incongruence.

- Self-blame for gender incongruence: Five parents reported blaming themselves for their child’s gender incongruence. Of these, one parent stated that he blamed himself for the child’s gender incongruence because, as a single and old parent, he had taken over the entire upbringing and the child had only ever been exposed to male influence, which might have led to the desire for a male gender identity. *“What did I do wrong? What went wrong?”* (group C, item 115).
- Self-blame for not noticing earlier: Two parents describe reproaching themselves for not noticing early signs of the child’s gender incongruence. *”And I felt like everything had slipped away from me*,* like I had done something wrong*,* like I should have noticed*,* that these were just excuses or something*,* I just couldn’t believe it. For me*,* it really felt like*,* ‘This can’t be true’. But I also remember that I thanked him for his honesty*,* for his trust*,* and I asked him how this could happen since everything seemed to have been going normally. He actually replied*,* ‘Yes*,* Mom*,* but you had enough other worries in the past few years*,*’ which*,* even today*,* still affects me because I thought*,* it’s crazy what a child goes through and holds back their own feelings*,* because they realize that other things consumed me so much that I didn’t have the ear for it. So*,* like I said*,* I thanked him*,* he went to bed*,* and honestly*,* I cried afterward.”* (group C, item 83).

## Discussion

**Fig. 5:**
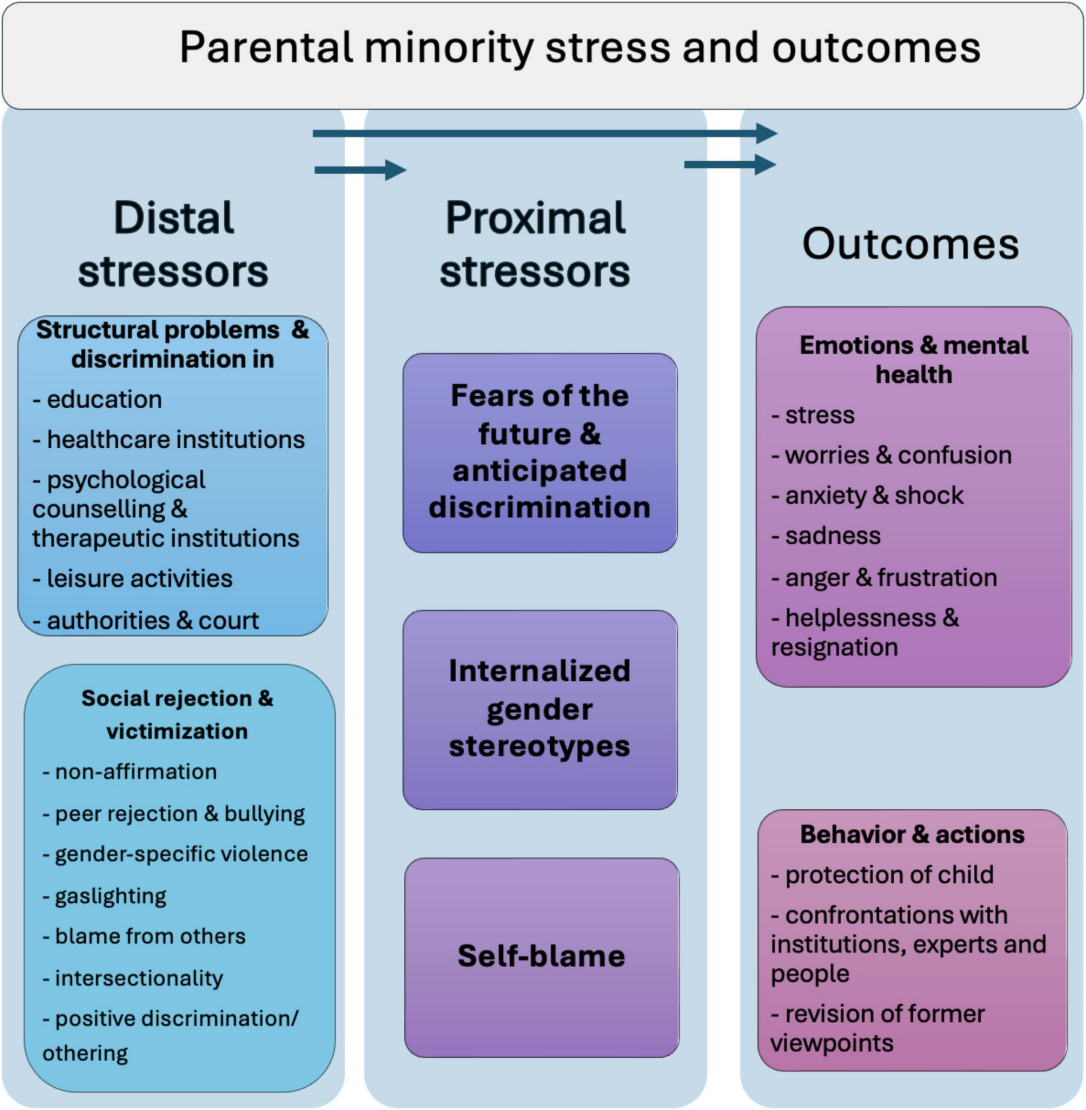
Contexts and content of parental minority stress and emotional and behavioral outcomes Source: Minority stress model adapted by Testa et al. [7]

This study aimed to analyze experiences of minority stress among parents of transgender adolescents. In focus groups, parents discussed aspects of minority stress they have been confronted and struggling with. Data were analyzed by QSCA and categorized as distal and proximal stressors according to the MSM adapted for transgender people [7]. Our findings align with and add to those of Hidalgo and Chen (2019) [25] who studied parents of younger, prepubertal children. Similarly, the parents in our study reported a broad spectrum of minority stress experiences, both directly as primary stressors and indirectly through their adolescent children as secondary stressors. Aligned to the models of Hidalgo and Chen [25] and Testa et al. [7] we summarized the findings in Fig. 5.

The results of this study contribute to a broader understanding of what it means for both pre- and peri-transitional transgender adolescents and their parents, belonging to a socially marginalized group.

Parents reported more distal than proximal stressors. The *distal stressors* could be divided into the overarching categories *structural burdens* and *social rejection*. Regarding *structural burdens*, the context of *education*, i.e. barriers to facilities, physical education and non-affirmative experiences like misgendering was most stressful for parents. The desperation of not being able to help the child with their school education and not getting support from teachers appeared to cause greater secondary minority stress than in other fields. An explanation could be that education shapes young people’s future, prospects and chances for a self-determined life which the parents perceive to be at risk. Based on evidence on parents’ critique about the educational system for causing minority stress for their transgender child and themselves [28, 47], researchers proposed concepts for inclusive transgender friendly schools. These, however, are still lacking in Germany and most countries [48]. In contrast to parents of pre-pubertal children [25], parents highlighted the lack of knowledge and expertise about gender incongruence among professionals, long waiting times, and insufficient information provided by healthcare providers. They expressed their frustration and helplessness asking: If not even professionals know what to do, who does? The burden observed in our study aligns with other studies in which parents reported that medical facilities, and therapeutic services are not adequately prepared to handle or support transgender youth [26, 49] and that access to both general and gender-affirming healthcare can be hindered by insufficiently trained personnel [27] beside the fact that there is a dramatic incline of waiting time within the mental health system worldwide [50]. These obstacles might fuel emotional stress among the parents [26]. With respect to the legal context, parents felt especially helpless when it came to the lacking support to change the child’s gender within the civil status. Recently in Germany in November 2024, the new Self-Determination Law came into effect, which allows transgender people to change their gender within the civil status without having to undergo psychological assessment according to the previous, highly stigmatizing and pathologizing “Transsexual Law” from 1980. While for minors counseling and parental consent is still required before changing the gender in legal terms [51], alleviating effects of this fundamental change in legislations towards affirmation can be anticipated, but need to be studied in future research. Also, parents expressed sadness that their child was afraid or had already experienced denial of access to *leisure and sport activities* because of their gender identity which is in line with other qualitative parent surveys [52, 53].

Another field of distal stressors was *overall social rejection*. Parents most frequently mentioned worries about *societal rejection*, like the lack of socio-cultural acceptance, growing trans-hostile political climate and anti-transgender propaganda. In the USA, numerous conservative states proposed bills to ban gender affirming care for minors, causing parents to even fear for their children’s life [54], and the current political developments seem to point toward a further deterioration of transgender rights not only in the USA but also in Germany [55]. These results show the great pressure parents are under and what kind of societal and political forces they must oppose to support their child. This is especially difficult when parents must combat their own *internalized gender stereotypes* as displayed in our findings, which is not only stressful for the parent but can also put a lot of pressure on transgender adolescents to perform in a cis-normative way [56]. Research on internalized gender stereotypes among parents of transgender children is limited, but related studies highlight the influence of societal norms on parental attitudes. Some parents struggle with internalized notions of traditional gender roles, leading to discomfort or stress in navigating their child’s gender identity [57, 58] and difficulty supporting their child’s non-binary identity due to binary norms which has been found in other parent surveys, too [52, 59]. More research is required on the needs of parents to feel empowered, especially when living in a trans-hostile social environment. Further, parents experienced stress and sadness because of their child’s *peer rejection* as reported as bullying or physical violence. Witnessing that their child’s well-being is at risk can be almost unbearable for parents and leads to immense distress [52, 59, 60]. Given the results presented so far, it is not surprising that the most frequently mentioned topic among proximal stressors was *fear of the future/anticipated minority stress* for their child. Parents are deeply concerned that their transgender child will not lead a happy life, will face discrimination in many areas of life, and will consequently suffer psychological distress. Enduring and ultimately addressing this fear is an important factor in the process of parental support during their child’s transition.

Further, parents experienced primary minority stress when being *gaslighted by other people* who denied their child’s trans identity which is why they feel that they must defend the child and themselves constantly. Gaslighting towards minorities is well documented in psychological research as a harmful form of emotional abuse [57, 61]. A study about gaslighting tactics towards LGBTQIA + people showed that making individuals doubt their perceptions can erode self-confidence and make victims feel isolated or “trapped,” often resulting in long-term psychological damage [62]. Research highlights its presence in private and institutional settings, reinforcing the need for awareness due to its far-reaching effects on mental health [63]. Noteworthily, parents of transgender adolescents have been neglected in this area so far. Another distal but primary stressor was the perception of *being blamed by others* for bad parenting or even being responsible for the child’s trans identity or mental health problems. These accusations and attacks can be very unsettling for parents and carry the risk that they internalize the blame [40, 64]. This aligns with our other findings, that parents identified self-blame as a proximal stressor too, feeling responsible for the difficulties their child is facing. Since feelings of guilt and shame are highly correlated with depression and anxiety [65] these experiences should be considered and consciously counteracted when providing care for families with transgender children. Another distal stressor identified was *intersectionality*, meaning the cross-over of various forms of discrimination (in this case racism, homo-hostility and trans-hostility). In line with our finding, a recent study with transgender adolescents’ parents of ethnic and sexual minorities found that families who experience various oppressions regarding their ethnicity, social and economic background, gender or disability have further needs of support compared to parents who are not affected by multiple discriminations [66]. However, more research with sufficient sample sizes and a focus on intersectionality is needed in the context of parenting transgender children. Another more subtle but therefore often overlooked form of minority stress which parents reported is *positive discrimination*. Parents noted that while the alleged intention is inclusion and support, i.e. when others emphasize the uniqueness of a transgender person, can reinforce *othering* where transgender adolescents are perceived as fundamentally different from their peers. Othering refers to the process of treating someone as “not normal” or “not belonging,” which may result in exclusion or stereotyping, stigmatization and could increase feelings of isolation which can extend to the parents, too [59, 67].

The *limitations* of this study include a small and selective sample since most of the parents surveyed had already demonstrated some level of support for their transgender children, with a relatively large number of adolescents already undergoing medical transition. However, there were also parents currently opposed to medical interventions who participated to exchange perspectives. Additionally, the focus group sessions were conducted only once and were time-limited, which constrained the depth of information collected and could have also led to parents being hesitant about sharing private information. This could also be reflected in the high imbalance between distal and proximal stressors, as parents may not have disclosed the extent to which they are personally affected. Also, there was a relatively high number of non-respondents in the questionnaires which may be explained by the fact that parents had already put in significant effort to participate in the focus groups, in some cases traveling long distances. Notably, the perceptions and answers of parents might have been influenced by various effects such as social desirability particularly in relation to the norms and perceptions of being a good parent and response biases due to time effects. Also, some experiences were only shared by one or two parents. These limitations must be considered in terms of transferability of the results. Furthermore, data collection took place during the COVID-19 pandemic, which may have amplified stressors for participants.

The qualitative results gained in this study suggest *implications* for gender affirming health care for pre- and peri-transitional transgender adolescents. Data showed that to a large degree, minority stress experiences of parents are closely intertwined with and relate to the minority stress experiences their transgender children are experiencing in everyday life [40, 68]. This finding highlights the importance of family-centered transgender health care with awareness for the multidimensionality of challenges each family member is experiencing. Specifically relating to parents of transgender adolescents, the study highlights new aspects of secondary stressors such as worries for the child’s education as well as parental primary stressors such as fears of the future, gaslighting and blame in terms of minority stress, adding to previous findings among comparable samples [25, 59, 67]. Identifying strategies to support parents in dealing with their everyday distal and proximal stressors, especially in the educational context and in the context of societal rejection as a parent of a transgender child, is crucial for ensuring they can offer the best possible support to their transgender children [14]. Notably, caution is required when extending the concept of minority stress to individuals who do not belong to marginalized groups themselves, as this carries the risk of downplaying the experiences of marginalized people. More research is needed to imbed our findings within a theoretical framework of an extended MSM. While we aimed to cover a broad range of aspects of parental minority stress, in-depth analyses of specific components will provide more detailed insights into parental experiences. Also, more education and knowledge is needed to identify more subtle forms of discrimination such as othering. In terms of intersectionality, transgender adolescents and their parents who belong to various marginalized groups, face challenges for which professional support should be specialized, which is often lacking in a cis- and heteronormative environment [11, 66]. Our findings can offer impulses for further research on the understanding of stress experiences which might hinder or enable parents to provide support, also regarding social and medical transition. Addressing these needs can facilitate both the parents’ adjustment and the adolescents’ transition processes, ultimately enhancing the well-being of both parties. It can be especially helpful here to recommend that parents connect with other parents of transgender children to support each other. Peer support among parents of transgender children has been shown to provide emotional and practical benefits, fostering resilience and reducing stress. Research highlights that such connections allow parents to share experiences, validate their feelings, and gain strategies for supporting their children effectively, improving family well-being [69]. Hence, there is further need for research to focus on resources and protective factors for parents of transgender children in the professional health care system.

## Conclusion

Some parents of trans children reported high sensitivity to minority stress they perceived for their children and experienced both distal and proximal stressors themselves. This stress may impact their emotional well-being, highlighting the need to support parents and caregivers in managing these challenges. Reducing minority stress for transgender individuals and their caregivers on a structural and inter-individual level should be a key target when thinking of inclusion and diversity. Interventions should focus on strengthening support systems for parents so they, in turn, can better support their children through the complexities of their transition process and overall journey.

## Electronic supplementary material

Below is the link to the electronic supplementary material.

Supplementary Material 1

## Data Availability

The datasets used and/or analyzed during the current study are available from the corresponding author on reasonable request.

## Footnotes

1 Transgender is used as an umbrella term for individuals who do not identify with their sex assigned at birth.

## Abbreviations

MSM: Minority Stress Model
QSCA: Qualitative Structuring Content Analysis
