# Supplementary material for: “And I don’t know how I can protect him from everything that’s coming” - A qualitative study on minority stress among parents of transgender adolescents
Source: BMC Psychiatry. 2025 Apr 24;25:419. doi: 10.1186/s12888-025-06822-3 (PMC12020220; doi:10.1186/s12888-025-06822-3)
Supplement: Supplementary file 1 — Supplementary Material 1 [file 12888_2025_6822_MOESM1_ESM.docx]

Supplementary Material

**S1** Semistructured interview guide for parents focus groups


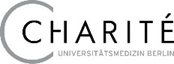


**Interview Guide for Focus Groups (Parents)**

**Part A – Gender Identity and Preferred Pronouns**

1. What brings you here today? Are you the parent or relative of a trans* child, or what is your relationship to the young person?
2. Is there anything that feels particularly important to you today?
3. How would you describe your child’s identity or gender expression?
4. What are their preferred pronouns, and how do others, as well as you, typically address them (most of the time/often)?
5. Is your child out? If yes, where and since when?
6. Would anyone like to share their experience of your child’s coming out?
7. Has your child begun their transition, and would anyone like to briefly share where they are in the process?
8. How are you currently feeling, and how have you felt in the past, regarding your child’s gender identity?

**Part B – Challenges**
9. Are there specific issues related to challenges that you would like to discuss?
10. What do you think are the biggest challenges regarding your child’s gender identity/gender expression?
• Within your family?
• Among their and your friends?
• In school/education?
• With institutions or authorities (e.g., public offices, postal services, etc.)?
• In the healthcare system, such as with doctors, psychologists, or insurance companies?
11. Does your child experience gender dysphoria? If so, how does it manifest, and how does it affect both your child and you?
12. Is there something about gender dysphoria that you wish others knew more about?
13. How do you currently feel about your child’s transition (if they are undergoing one)?
14. Is there something about the transition process that you wish others understood better?
15. What do you consider to be the biggest challenge, both currently and in the past?
16. When you feel burdened or sad, who do you turn to? Who does your child turn to?


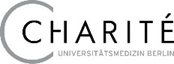


**Part C – Resources**
17. When you feel burdened or sad, is there anything that helps your child? What helps you?
18. Where does your child feel accepted in their identity/gender expression? What makes them feel this way?

19. Where do you feel understood and accepted? What are your safe spaces?
20. What makes you and your child particularly happy?

**Part D – Needs**
21. What would further help your child live their identity/gender expression more openly and accepted? What do you wish for in this regard from...
• Parents, siblings (family)?
• Friends?
• School, education?
• Institutions, authorities?
• Healthcare system, especially concerning transition?
22. In your opinion, is there anything that should change in these areas and in society at large?
23. Is there something you would like to share with other parents of trans* children?
24. What do you wish for your child?
25. What do you wish for from doctors and psychologists?

**Closing Questions:**
26. Are there any research questions you would have liked to discuss?
27. What topics should research supporting trans*, non-binary, and genderqueer people focus on, in your opinion?

28. How did you like the focus group?
29. Any final remarks?
